# Grandparent Resilience: Improving Self-Efficacy in Grandparents Raising Grandchildren

**DOI:** 10.1093/geroni/igaa057.1647

**Published:** 2020-12-16

**Authors:** Aimee Fox, Nathaniel Riggs, Loriena Yancura, Christine Fruhauf

**Affiliations:** 1 Colorado State University, Fort Collins, Colorado, United States; 2 University of Hawai’i, Honolulu, Hawaii, United States

## Abstract

Grandparents often protect against childhood trauma and promote resilience through their nurturance, love, and support when raising grandchildren. Despite the beneficial role grandparents have on their grandchildren, grandparents may experience challenges of their own, including physical, mental, and emotional health issues, lack of resources, and social isolation. Few interventions exist to help grandparents successfully adapt to the challenges they face as primary parenting figures. The purpose of this study was to test preliminary efficacy of a strengths-based intervention for grandparents raising grandchildren aimed at increasing self-care behaviors, managing emotions, and connecting to community resources. Grandparents (N = 137) providing primary care to grandchildren were recruited to participate in a single-group, pre- and post-test design, 6-week intervention. Self-efficacy was assessed at baseline, post-intervention, and at a 6-month follow-up. To evaluate who the intervention might be most beneficial for, grandparents’ service knowledge, perceived support from others, and length of care provided, measured at baseline, were analyzed for moderating effects. Results of paired-samples t-tests reveal significant increases in self-efficacy (p = .013) from baseline to post-test, which were maintained at the 6-month follow-up (p = .010). Hierarchical multiple regression showed interaction effects of the hypothesized moderators were not significant, indicating improvements in self-efficacy regardless of individual variability at baseline. As demonstrated, interventions can be effective at increasing self-efficacy in grandparents raising grandchildren and strengths-based approaches have the potential to provide universal benefits to grandparents, thus improving functioning in grandfamilies and promoting the health and well-being of grandparents and their grandchildren.

